# Survey of Pediatric Cardiologists on Screening for Conditions Associated with Sudden Cardiac Death

**DOI:** 10.1007/s00246-025-03987-2

**Published:** 2025-08-14

**Authors:** Jenna L. Schlondrop, Nicholas H. Von Bergen, Xiao Zhang, John S. Hokanson

**Affiliations:** 1 University of Wisconsin-Madison Undergraduate Research Scholars Program, Madison, USA; 2 Division of Pediatric Cardiology, Department of Pediatrics, University of Wisconsin-Madison School of Medicine and Public Health, H6/516C, 600 Highland Ave., Madison, WI 53792 USA

**Keywords:** Sudden Cardiac Death, Preparticipation Screening, Pediatric Cardiology, Pediatric Electrophysiology

## Abstract

Sudden cardiac death (SCD) in children and adolescents is rare but receives considerable attention due to its tragic nature. Even so, guidelines, provider practice and opinion regarding pre-participation screening for conditions associated with SCD in young competitive athletes and non-athletes remain varied. We sought to determine the recommendations on this topic from practicing pediatric cardiology providers. An anonymous survey regarding pediatric cardiovascular providers’ opinions and practices on the screening of high school aged youth for conditions associated with SCD was collected from the American Academy of Pediatrics Section on Cardiology and Cardiac Surgery and the Pediheart.net online community. Responses were evaluated and compared between pediatric electrophysiologists and all other respondents. A slight majority of respondents (56.8%) believe that pre-participation screening for conditions associated with SCD high school age individuals should not be different based on one’s status as a competitive athlete. Respondents felt that the current emphasis on pre-participation screening for competitive high school athletes varied with respondents considering it too little in 26.1% appropriate in 42.2% and too much in 31.7%. Our survey reflects the clinical recommendations of practicing pediatric cardiologists and pediatric electrophysiologists. Overall, our data does not demonstrate a consensus on the role of ECG for pre-participation screening, and there is no obvious desire for increased universal screening of high school aged individuals among pediatric cardiology providers.

**Supplementary Information:**

The online version contains supplementary material available at 10.1007/s00246-025-03987-2.

## Introduction

The sudden cardiac death (SCD) of a high school student is a tragic but rare event, occurring somewhere between 4.6 and 15.2 per million high schoolers per year occur [1–6]. SCD is defined as death from unanticipated cardiovascular causes that occurs within 1 h of the onset of symptoms, or within 24 h of symptom onset if the case was not witnessed [7]. The incidence varies in different populations and may be as much as fourfold higher in high school boys (22.7 per million person years) than in girls (4.9 per million person years) [6]. There may also be racial differences in the incidence of SCD with African American college athletes suffering a higher rate of SCD [8]. Additionally, screening tests may also have variable predictive value, such as with male African American basketball players who are more likely to have abnormal pre-participation screenings than other high school athletes (10).

The decision-making regarding pre-participation screening in high school aged individuals for conditions associated with SCD remains controversial and no clear consensus is present between various organizations. The American Academy of Pediatrics (AAP) recommends a pre-participation physical for young athletes but does not recommend routine ECG screening (11). Recently, the American Heart Association and American College of Cardiology issued guidelines on pre-participation screening which describe the use of resting ECG as reasonable so long as the ECG is interpreted appropriately and systems are in place for downstream clinical evaluation (12).

As many of the conditions thought to predispose a high school age individual to SCD are cardiac in nature, pediatric cardiologists and pediatric electrophysiologists (EP) are often considered experts in the diagnosis and management of these conditions. Although many authors and organizations have made recommendations on the optimal screening for SCD in young people, there has been no systematic assessment of what practicing pediatric cardiovascular experts recommend for their patients. Therefore, this manuscript evaluates the recommendations given by pediatric cardiologists including pediatric electrophysiologists.

## Methods

The University of Wisconsin Minimal Risk Institutional Review Board reviewed and approved this study. An anonymous electronic survey was created using Qualtrics software to assess the current opinions and practices of pediatric cardiologists regarding screening for conditions associated with sudden cardiac death. The survey consisted of 33 questions addressing individual and place of practice information, respondents’ perception of screening, respondents’ recommendations for screening (Supplemental Fig). Surveys were considered for inclusion in this report if the respondents reached the key question on the appropriateness of screening. The survey was reviewed before distribution by the American Academy of Pediatrics Section on Cardiology and Cardiac Surgery (AAP SOCCS).

In the spring of 2023 recruitment emails were sent to approximately 1,000 members of AAP SOCCS and 1,500 members of the Pediheart community, with considerable overlap between the groups. As the question of screening for conditions associated with SCD most commonly arises in the context of adolescents (13), our report focuses on the survey responses related to high school age children (ages 14–18 years).

Questions included queries about differences in practice recommendations between competitive athletes and those who were not considered competitive athletes. A competitive athlete was defined as “one who participates in an organized team or individual sport that requires regular competition against others as a central component, places a high premium on excellence and achievement and requires some form of systematic (and usually intense) training” consistent with the definition in prior guidelines (11, 12).

Simple descriptive statistics were calculated on demographic factors and practices to screen for conditions associated with SCD. Chi square test was used to compare responses between pediatric electrophysiologists and all other respondents. Paired t test was performed to compare estimates of SCD risks for high school male athletes versus their female counterparts. All analyses were performed with the statistical software STATA/SE 16.1 (StataCorp, College Station, TX). A *p*-value of 0.05 was considered as significant.

## Results

A total of 206 individuals opened the survey, of which 192 completed the demographics section. 142 respondents answered key questions regarding the appropriateness of the emphasis placed on screening. We report the responses of these 142 individuals. Their demographics are provided in Table 1.

**Table 1:** Respondent Demographics

Respondents	142
Attending Cardiologists	114
Mean Years of Practice	17
(Attending Cardiologists)	
Read ECGs	138
Electrophysiologists	21
Region	
Central Plains/Rockies	4
West Coast	14
Northeast	34
Midwest	39
South	41
Program Annual Surgical Volume	
Non-Surgical	19
Less than 150	22
150–300	50
More than 300	48

## Demographics

Of the 142 respondents that answered the key questions, 114 were attending cardiologists with an average of 17 years in practice. There were 21 electrophysiologists included in the responses. Others that responded to the survey included advanced practice providers and trainees. Most respondents read screening ECGs.

## Estimates of SCD in High School Athletes

Respondents were asked to estimate the incidence of sudden cardiac death in high school age boys and girls. The results are provided in Table 2 and compared with existing literature. Our respondents’ overestimated the risk for SCD in high school girls and underestimated the risk of SCD in high school boys, which has been reported to be significantly higher (22.7/million person years) than girls (4.9 per million person years) (6).

**Table 2:** Respondents’ Estimates of SCD in Events Per Million Person Years

Population in Question	Mean	IQ Range	Literature (6)
High School Boys	13.4	5 to 15	22.7
High School Girls	10	3 to 10	4.9

## Who to Screen

In general, respondents did not recommend altering screening based on race and ethnicity (89%) or gender (93%). However, a significant minority of respondents (43.2%) believe that pre-participation screening for conditions associated with SCD should be different for competitive athletes compared to those who were not competitive athletes (Table 3).

**Table 3:** Demographic Criteria Which Should be Considered When Screening for Conditions Associated with Sudden Cardiac Death

	All Respondents	Pediatric Electrophysiologists	All Other Respondents	*P* value
Gender	1/136 (7.4%)	1/20 (5%)	9/116 (7.8%)	1
Race	15/140 (10.8%)	3/20 (15%)	12/120 (10%)	0.454
Status as a Competitive Athlete	60/139 (43.2%)	4/20 (20%)	56/119 (47.1%)	0.028

When evaluating the recommendations of non-pediatric electrophysiologists in comparison to the pediatric EP providers, the non-EP providers were statistically more likely to recommend considering the status as a competitive athlete than the EP providers (47% of non-EP vs 20% of EP providers) for screening. There was no statistically significant difference in responses from the 21 pediatric electrophysiologists and 121 other pediatric cardiologists regarding whether screening should differ based on youth’s race or gender.

## Respondent Screening Recommendations

Regarding their individual recommendations for the screening of high school-age individuals, 41% of respondents recommended ECGs for competitive athletes, and 22% recommended ECGs for those who are not identified as competitive athletes (Table 4). Echocardiography was recommended for competitive athletes by 9% of respondents, but only 2 respondents recommended echocardiographic screening for those who are not identified as competitive athletes. There was no statistically significant difference in the screening recommendations from electrophysiologists and the other respondents.

**Table 4:** Screening Tests Recommended Based on Athletic Status

	All Respondents	Pediatric Electrophysiologists	All Other Respondents	*P* value
I recommend ECG for HS Competitive Athletes	57/139 (41%)	5/20 (25%)	52/119 (43.7%)	0.116
I recommend ECG for HS who are not Competitive Athletes	3/139 (21.6%)	6/20 (30%)	24/119 (20.2%)	0.323
I recommend Echo for HS Competitive Athletes	13/139 (9.4%)	2/20 (10%)	11/119 (9.2%)	1
I recommend Echo for HS who are not Competitive Athletes	2/139 (1.4%)	1/20 (5%)	1/119 (0.8%)	0.268

## Appropriateness of Screening an Liability Concerns

**Fig. 1:**
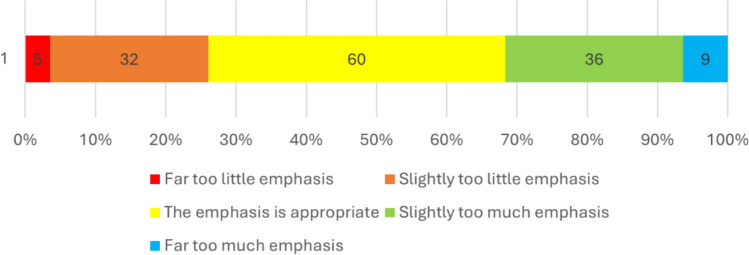
Respondents’ Perception of the Current Emphasis on Screening for Conditions Associated with Sudden Cardiac Death

Respondents generally thought the emphasis on screening was appropriate (Fig. 1). Approximately 1/3 of respondents (31.7%) thought that there was too much (anywhere from slightly to far too much) emphasis on screening. While 26.1% thought that the emphasis on screening was too little (from slightly or far too little).

**Fig. 2:**
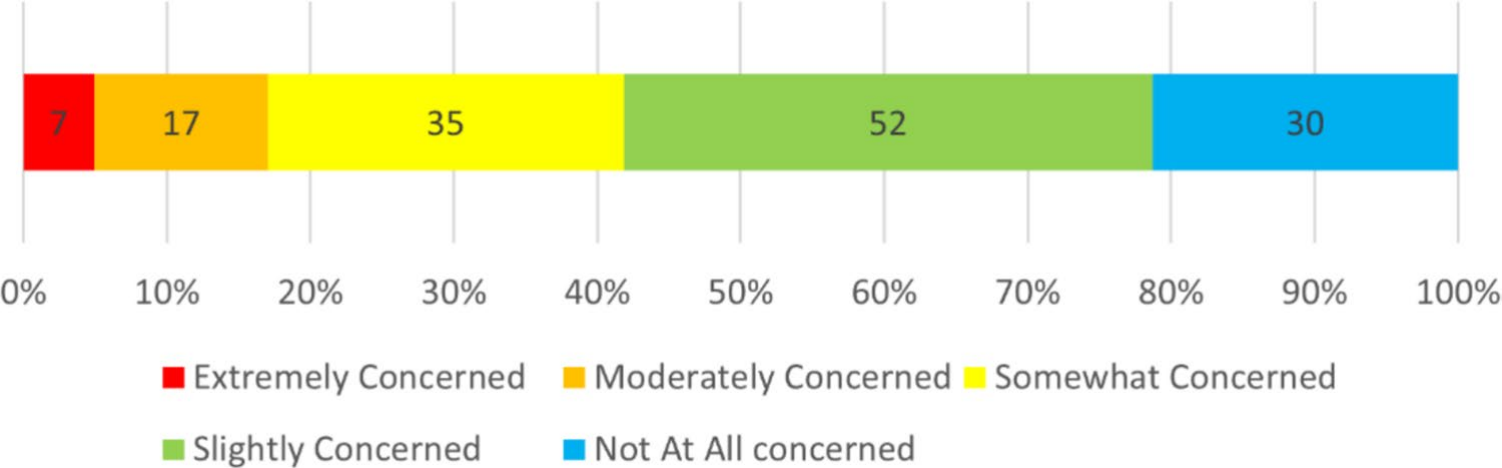
Respondents’ Level of Concern for Liability When Reading Screening ECGs

Concerning liability when reading ECGs, 16% of respondents were at least moderately concerned about the liability associated with reading screening ECGs, and 4% of those respondents were extremely concerned. (Fig. 2).

## Discussion

This survey assessed provider perceptions of sudden cardiac death (SCD) risk, screening practices for conditions associated with SCD, and current approaches to caring for high school aged patients. While SCD in children is a tragic event, we also acknowledge it is also a rare event, with more articles focused on SCD each year, including this study, than actual SCD in youth in competitive athletes. Although there is a wealth of recommendations from individual authors and organizations to our knowledge, this study represents the first assessment of what pediatric cardiovascular providers recommend in clinical practice.

Overall, the results of the survey do not support the practice of substantial additional screening in most high school students, with most providers recommending only routine physical examination and risk assessment questionnaire.

Most respondents did not feel that screening should be altered due to gender or race despite the data demonstrating differences in the performance of SCD screening (10) and the incidence of SCD in different populations (6, 8). The results provided in Table 2 suggest that our respondents have not included the data on differences in the incidence of SCD by sex (6) which may have influenced their recommendations. It is also possible that idea of providing differing levels of screening based on race and gender may itself have been unpalatable to respondents. Many more respondents felt that screening should be altered based on designation as a competitive athlete. This was particularly true of respondents who were not pediatric electrophysiologists, who were more than twice more likely to recommend ECG screening for athletes than non-athletes. Overall, respondents recommended an ECG in 41% and an echocardiogram in 9% of competitive athletes in comparison to 22% and 1% in those who are not competitive athletes. We appreciate that SCD is not restricted to competitive athletes and much of the published data cannot be generalized for the entire population.

This survey did not assess who would perform the screening tests, the potential for false-positive or false-negative results, or the associated costs of screening and subsequent testing. Unfortunately, the important topic of health equity of those screened was beyond the scope of this survey. Although minority of respondents reported moderate or greater concern about liability when interpreting ECGs in this setting, only 21.1% reported that they were unconcerned about this possibility.

There were several limitations to this survey. Although this data provide some insight into the recommendations of practicing pediatric cardiologists and pediatric electrophysiologists, the small sample size may be underpowered to detect important distinctions between pediatric cardiologists and electrophysiologists. Due to considerable overlap between members of AAP SOCCS and Pediheart we cannot calculate a response rate or evaluate for duplicate responses. Only a small number of EPs (*n* = 21) completed the survey. The results from this survey represent a minority of those who were invited and not everyone answered every question. Although the respondents represent a broad range of practice settings and geographic locations, we acknowledge the respondents do not necessarily represent the pediatric cardiology community as a whole. By definition, this study includes a sample bias toward those providers willing to participate in an online survey. Our results may also be skewed toward those with the strongest opinions on the topic.

## Conclusion

This study is the first to describe current cardiac screening recommendations made by practicing pediatric cardiologists and electrophysiologists. Our findings demonstrate a lack of consensus on pre-participation screening, and most respondents do not routinely recommend either an ECG or echocardiogram for pre-participation evaluation. Respondents thought that screening for conditions associated with SCD should not be altered based on gender or race, but a notable minority felt someone’s status as a competitive athlete should influence screening practice.

With the exception of the role of athletic status in screening, pediatric electrophysiologists did not have significantly different responses to the survey than other respondents, but the sample size was too small to draw definite conclusions. Overall, our survey does not suggest broad support among pediatric cardiology specialists for expanding cardiac screening in high school aged adolescents.

## Supplementary Information

Below is the link to the electronic supplementary material.

Supplementary file1 (PDF 326 KB)

## Data Availability

No datasets were generated or analysed during the current study.

## Abbreviations

AAP SOCCS: American academy of pediatrics section on cardiology and cardiac surgery
AHA: American heart association
ECG: Electrocardiogram
EP: Electrophysiologist
SCD: Sudden cardiac death
