# Correction: Survey of Pediatric Cardiologists on Screening for Conditions Associated with Sudden Cardiac Death

**DOI:** 10.1007/s00246-025-04011-3

**Published:** 2025-09-12

**Authors:** Jenna L. Schlondrop, Nicholas H. Von Bergen, Xiao Zhang, John S. Hokanson

**Affiliations:** 1 University of Wisconsin-Madison Undergraduate Research Scholars Program, Madison, USA; 2 Division of Pediatric Cardiology, Department of Pediatrics, University of Wisconsin-Madison School of Medicine and Public Health, H6/516C, 600 Highland Ave., Madison, WI 53792 USA

**Correction to: Pediatric Cardiology** 10.1007/s00246-025-03987-2

In this article reference from 9–13 was missing and should have been read as below:

9. Corsi DR, Kelly B, Nair N, Luo M, Osler B, Cho S–H, Mehrotra P, Wiener D, Johnson D (2025) Cardiac screening findings and referral patterns in male African-American basketball players: analysis of the HeartBytes Registry. Am J Cardiol 243:73–80

10. Corsi D, Saraiya A, Doyle M et al (2025) Cardiac screening findings and referral patterns in male African-American basketball players: analysis of the HeartBytes Registry. Am J Cardiol 243:73–80. 10.1016/j.amjcard.2025.02.007

11. Kinsella S (2024) Preparticipation physical evaluation. Pediatr Care Online. 10.1542/aap.ppcqr.396018 (Published 17 April 2024)

12. Kim JH, Baggish AL, Levine BD et al (2025) Clinical considerations for competitive sports participation for athletes with cardiovascular abnormalities. J Am Coll Cardiol 85(10):1059–1108. 10.1016/j.jacc.2024.12.025

13. Westaby JD, Sheppard MN (2025) Sudden cardiac death in childhood: peaks in teenagers. Circ Arrhythmia Electrophysiol 18(2). 10.1161/CIRCEP.124.013355

The original article has been corrected.

