# Supplementary material for: Survey of Pediatric Cardiologists on Screening for Conditions Associated with Sudden Cardiac Death
Source: Pediatr Cardiol. 2025 Aug 14;47(5):2002–6. doi: 10.1007/s00246-025-03987-2 (PMC13144251; doi:10.1007/s00246-025-03987-2)
Supplement: Supplementary file 1 — Supplementary file1 (PDF 326 KB) [file 246_2025_3987_MOESM1_ESM.pdf]

WISCONSIN
UNIVERSITY OF WISCONSIN–MADISON

Consent and Demographics

A Survey on Screening for Conditions Associated with Sudden Cardiac Death

My name is Jenna Schlondrop, and I am an undergraduate student at the University of Wisconsin-Madison studying nursing. I am performing a study on screening for sudden cardiac death in young athletes with an emphasis on the use of ECG.

I would be grateful if you would complete the attached on-line study.

This study should take less than 10 minutes to complete. Your responses to the survey will remain confidential.

Participating in this study is completely voluntary. You are free to skip any survey questions that you do not want to answer. Even if you start the survey, you are not required to

complete it, and can stop at any time. The survey is anonymous, and no one associated with the study will be able to link your responses back to you. Please do not include your name or any other identifying information in your survey responses. This study was approved by the University of Wisconsin Health Sciences Minimal Risk Institutional Review Board.

I intend to present the results of this survey at the 2023 American Academy of Pediatrics National Conference and Exhibition during the sessions of the Section on Cardiology and Cardiac Surgery.

My mentor for this study is Dr. John Hokanson in the Department of Pediatrics. Please contact them with questions about this study at 608-262-5024.

☐ I agree to participate in this on-line survey

Thank you for participating in this study. Even if you have received more than one invitation to this survey, please complete it only once.

If you have any technical questions regarding this survey,

please contact the information technology staff at the University of Wisconsin at help@pediatrics.wisc.edu.

In this survey we will use the same definition of a **competitive athlete** found in the AHA/ACC Scientific Statement on Eligibility and Disqualification Recommendations for Competitive Athletes With Cardiovascular Abnormalities as:

One who participates in an organized team or individual sport that requires regular competition against others as a central component, places a high premium on excellence and achievement, and requires some form of systematic (and usually intense) training.

- Got it

What is your gender identity

- Male
- Female
- Non-binary / third gender
- Prefer not to say

What is your field of practice?

- General Pediatric Cardiology
- Pediatric Electrophysiology
- Other Pediatric Cardiology
- Pediatric Cardiac Surgery
- Pediatric Intensive Care
- [ ] Other

What is your role?

- Attending Physician
- Advanced practice provider
- Trainee
- [ ] Other

How many years have you been practicing?

[ ]

In which country is your primary practice?

- United States
- Canada
- Mexico
- [ ] Other

If United States, please specify region.

[ ]

What is the annual cardiac surgical volume of your practice?

- Non-surgical practice
- Less than 150 cases/year
- 150-300 cases/year
- More than 300 cases/year
- I don't know

ECG Screening Questions

Do you read Electrocardiograms (ECGs)?

- Yes
- No

Do you read pre-participation screening ECGs in the same way as other ECGs?

- I read screening ECGs in the same way as other ECGs
- I read screening ECGs differently than I read other ECGs
- I do not read screening ECGs

Do you use the Seattle criteria when reading pre-participation screening ECGs in high school athletes (ages 14-18)?

- Yes
- No
- I do not read screening ECGs

What is your level of concern about the liability cardiologists face when reading pre-participation

screening ECGs?

- Not at all concerned
- Slightly concerned
- Somewhat concerned
- Moderately concerned
- Extremely concerned

Please estimate the percentage of **false positive** results on pre-participation ECGs for conditions associated with sudden cardiac death in these age groups.

	Percentage<br>0 10 20 30 40 50 60 70 80 90 100	I don't know	
Elementary School (ages 6-10)		☐	
Middle School (ages 11-13)		☐	
High School (ages 14-18)		☐	

Please estimate the percentage of **false negative** results on pre-participation ECGs for conditions associated with

sudden cardiac death **that should be detected with an ECG** in these age groups.

	Percentage (0 10 20 30 40 50 60 70 80 90 100)	I don't know	
Elementary School (ages 6-10)		☐	
Middle School (ages 11-13)		☐	
High School (ages 14-18)		☐	

General Questions

How would you rate the emphasis placed on pre-participation screening of children and adolescents for conditions associated with sudden cardiac death?

- Far too little emphasis
- Slightly too little emphasis
- The emphasis is appropriate
- Slightly too much emphasis
- Far too much emphasis

What is the incidence of sudden cardiac death in elementary, middle, and high school-aged **MALES**? (please estimate cases per million patient-years)

	(enter cases per million patient-years) 0 10 20 30 40 50 60 70 80 90 100	I don't know	
Elementary School (ages 6-10)		☐	
Middle School (ages 11-13)		☐	
High school (ages 14-18)		☐	

What is the incidence of sudden cardiac death in elementary, middle, and high school-aged **FEMALES**? (please estimate cases per million patient-years)

	(enter cases per million patient-years) 0 10 20 30 40 50 60 70 80 90 100	I don't know	
Elementary School (ages 6-10)		☐	
Middle School (ages 11-13)		☐	
High School (ages 14-18)		☐	

What is your preferred age range to perform pre-participation screening for conditions associated with sudden death?

- ◯ Elementary school (ages 10 and under)
- ◯ Middle school 11-13)
- ◯ High school (ages 14-18)
- ◯ Post high school (ages 18-21)
- ◯ I do no have a preference

Should screening for conditions associated with sudden cardiac death be different for competitive athletes compared to other patients?

As stated earlier, for this survey we define a competitive athlete as one who participates in an organized team or individual sport that requires regular competition against others as a central component, places a high premium on excellence and achievement, and requires some form of systematic (and usually intense) training.

- ◯ Yes
- ◯ No

What type of screenings should be done for **competitive high school athletes**? Please check all that apply.

- ☐ Standard well child check
- ☐ AHA 14-point history and physical
- ☐ ECG
- ☐ Echocardiogram

What type of screenings should be done for **high school students who do not identify as competitive athletes**? Please check all that apply.

- ☐ Standard well child check
- ☐ AHA 14-point history and physical

- ☐ ECG
- ☐ Echocardiogram

What type of screenings should be done for **high school students**? Please check all that apply.

- ☐ Standard well child check
- ☐ AHA 14-point history and physical
- ☐ ECG
- ☐ Echocardiogram

Should pre-participation screening for conditions associated with sudden cardiac death be done differently based on race or ethnicity?

- ○ Yes
- ○ No

What races/ethnicities should be emphasized in screening? Please select all that apply.

- ☐ Black or African American
- ☐ American Indian or Alaskan Native
- ☐ Asian

- ☐ Native Hawaiian or Other Pacific Islander
- ☐ White
- ☐ Middle Eastern and North African
- ☐ Hispanic and Latino/a
- ☐ [ ] Other

Should preparticipation screening for conditions associated with sudden cardiac death be done differently based on sex?

- ○ Yes
- ○ No

Which sex should have more emphasis on screening?

- ○ Male
- ○ Female

Which entities require pre-participation screening in your community? Please check all that apply.

- ☐ School districts
- ☐ Health care system

- ☐ State government
- ☐ University Athletic Programs
- ☐ State Athletic Associations
- ☐ None
- ☐ [ ] Other
- ☐ I don't know

Who pays for the follow-up testing of abnormal findings?

- ☐ Patient/Family
- ☐ Parent or community organization
- ☐ School districts
- ☐ [ ] Other
- ☐ I don't know

Please estimate the percent of subjects who complete subsequent testing if their pre-participation screening is abnormal.

	0	10	20	30	40	50	60	70	80	90	100	I don't know	
Percent completing subsequent testing												☐	☐

Are mass pre-participation screenings of athletes performed in your community?

- ○ Yes
- ○ No
- ○ I don't know

Who organizes these mass screenings?

- ○ Parent or community organization
- ○ Health care systems
- ○ School districts
- ○ ☐ Other
- ○ I don't know

Are ECGs a part of these mass screenings?

- Yes
- No
- I don't know

Who reads the ECGs if they are a part of these mass screenings?

- Pediatric Cardiologist, Volunteer
- Pediatric Cardiologist, Paid
- Other Physician, Volunteer
- Other Physician, Paid
- Other individual, Volunteer
- Other individual, Paid
- I don't know

What is the socioeconomic status of the communities in which these mass screenings are occurring?

- High income community
- Middle income community
- Low income community
- Wide mix of incomes
- I don't know

Powered by Qualtrics
